# Changes in Joint Gap Balances between Intra- and Postoperation in Total Knee Arthroplasty

**DOI:** 10.1155/2014/790806

**Published:** 2014-01-02

**Authors:** Arata Nakajima, Yasuchika Aoki, Masazumi Murakami, Koichi Nakagawa

**Affiliations:** ^1^Department of Orthopaedic Surgery, Toho University Sakura Medical Center, 564-1 Shimoshizu, Sakura, Chiba 285-8741, Japan; ^2^Department of Orthopaedic Surgery, Chiba Aoba Municipal Hospital, 1273-2 Aoba-cho, Chuo-ku, Chiba 260-0852, Japan

## Abstract

Achieving correct soft tissue balance and preparing equal and rectangular extension and flexion joint gaps are crucial goals of TKA. Intraoperative gap balances would change postoperatively; however, changes in joint gap balances between pre- and postoperation remain unclear. To explore these changes associated with TKA, we prospectively investigated 21 posterior cruciate ligament retaining TKAs for varus knees. Intraoperative extension gap balance (iEGB) was 2.6 ± 2.0° varus versus postoperative extension gap balance (pEGB) of 0.77 ± 1.8° valgus (*P* < 0.01), while no significant difference between intraoperative flexion gap balance (iFGB) and postoperative flexion gap balance (pFGB) was observed. We also explored correlations between intraoperative and postoperative gap balances but found no significant correlations. These observations indicate that (i) surgeons should avoid excessive release of the medial soft tissue during TKA for varus knees and (ii) intraoperative gap balance may not be necessarily reflected on postoperative gap balance.

## 1. Introduction

Achieving correct soft tissue balance of the knee is fundamental to the success of TKA [1], and an equal joint gap during extension and flexion is a prerequisite for satisfactory soft tissue balance [2–4]. In addition, equalizing the distance from the femoral component to the tibial surface (i.e., the joint gap) throughout the full range of knee motion prevents lift-off of the tibial component and theoretically assists in achieving proper contact pressure and kinematics. Thus, preparing equal and rectangular extension and flexion joint gaps is the most important goal of TKA.

Meanwhile, most surgeons agree that accurate ligament balancing of the knee with varus deformity is difficult especially during posterior cruciate ligament retaining (CR)-TKA. The standard procedure for ligament balancing of the medial side of the knees uses subperiosteal release of the medial collateral ligament (MCL) [5–7]. Despite performing such release of MCL, varus balance is likely to remain in most CR-TKAs because posterior cruciate ligament (PCL) is an important component of the medial supporting mechanism of the knee [8].

Recently, Sekiya et al. reported that residual lateral ligamentous laxity immediately after surgery subsequently corrected itself spontaneously in some instances [9]. This finding suggests that some degree of residual lateral laxity, namely, varus balance, may be tolerable for varus knees so long as proper valgus alignment is maintained. However, it has not been explored whether remaining varus balance is also tolerable for CR-TKAs where intraoperative varus balance is likely to remain.

Sasanuma et al. compared soft tissue balance during TKA with soft tissue balance after TKA and concluded that immediate postoperative coronal laxity correlated positively with intraoperative coronal laxity at 0° (extension) but exhibited no correlation with intraoperative coronal laxity at 90° (flexion) [10]. So far, however, relationship between intraoperative and postoperative gap balance in TKA has not been elucidated fully.

In this study, we hypothesized that intraoperative varus balance would improve postoperatively in CR-TKAs and that intraoperative gap balance would correlate with postoperative gap balance. To verify these hypotheses, we prospectively investigated the posterior cruciate ligament retaining (CR)-TKAs for varus-deformed knees. We measured intraoperative tibiofemoral gap balance in knee extension and flexion before implanting femoral and tibial components and consecutively measured tibiofemoral gap balance four to six weeks postoperatively. We then used these measurements to analyze the relationship between intraoperative and postoperative gap balances.

## 2. Patients and Methods

We analyzed 21 selected knees in 20 patients with osteoarthritis (4 men and 16 women with a mean age of 75.0 years, ranging from 61 to 82 years) who had undergone TKA in Chiba Aoba Municipal Hospital between June 2009 and February 2010. Knees with ankylosis or severely limited range of motion (fixed flexion deformity >20 or flexion <90) were excluded from this study. All patients received a CR-type Scorpio NRG (Stryker Orthopedics, Mahwah, NJ).

**Figure 1:**
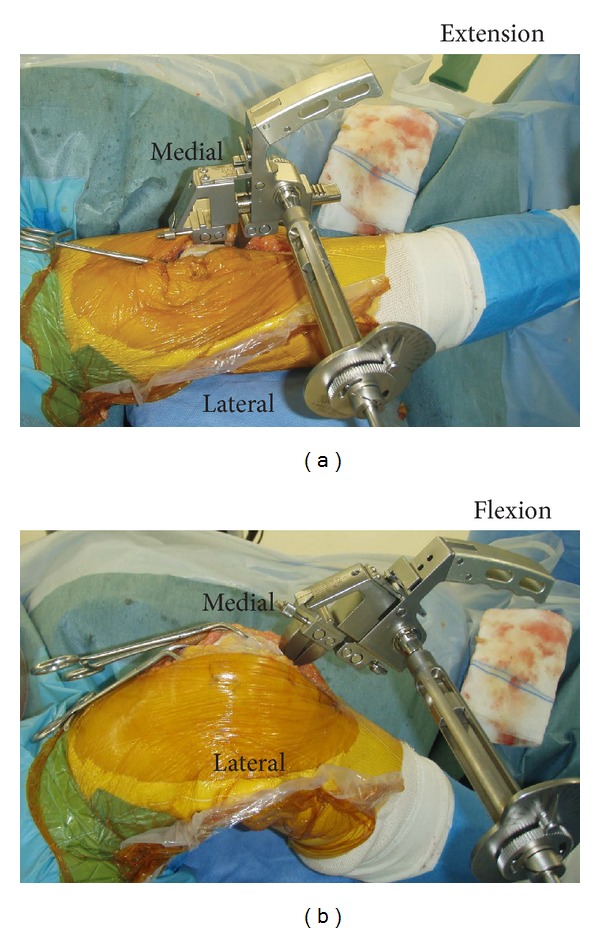
Measurements of intraoperative gap balance for the right knee at 0° (extension) and 90° (flexion) with the patella in a reduced position and a constant 40-pound distracting force being applied between the cut surfaces of the distal femur and the proximal tibia.

We performed all operations in accordance with previously published descriptions of the gap technique [11–13] with our modifications. After bone cuts, we measured the joint gap and medial and lateral soft tissue balance at 0° and 90° flexion with the patella reduced (intraoperative gap balance), using a tensor device (Stryker Orthopedics) (Figure 1). We applied a constant 40-pound distracting force between upper and lower plates via a ratchet-type hex wrench which limited the applied force to 40 pounds [14, 15]. Finally, we added medial soft tissue release for all cases according to the staged release reported by Clayton et al. [16] until both extension and flexion gap balances would be within 5° and then recorded the measurements as intraoperative gap balances. We implanted appropriately sized femoral and tibial components along with a polyethylene insert of the appropriate thickness.

**Figure 2:**
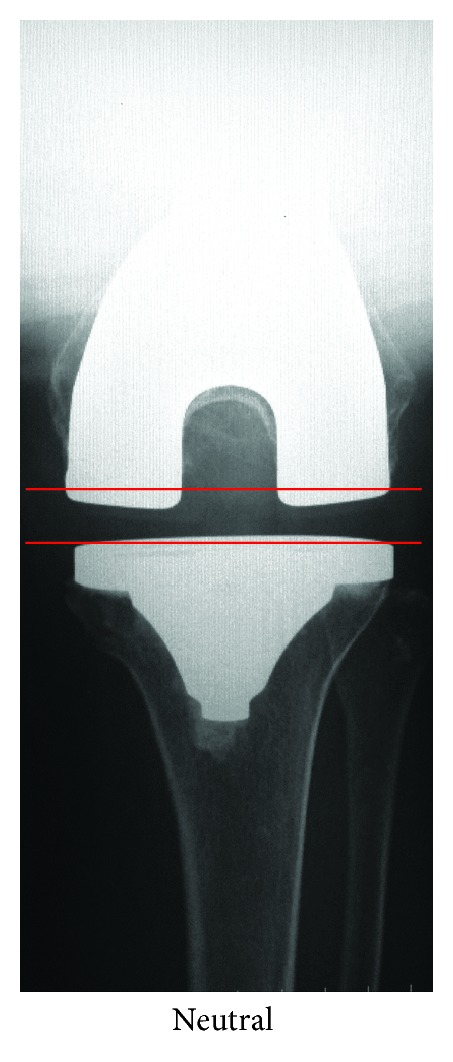
Postoperative axial radiographs of the left distal femur. Patients sit on a table with their lower legs dependent and a 1.5 kg weight attached to the ankle on the treated side. The angle between the cut lines of the posterior femoral condyles and the proximal tibia represents the flexion gap balance (FGB).

Four to six weeks after surgery, we evaluated medial and lateral soft tissue balance at knee extension and flexion positions (postoperative gap balance) in a knee-neutral position. In extension, we took anteroposterior radiographs with patients in a supine position and measured the angle between the cut lines of the distal femur and the proximal tibia. In flexion, we took axial radiographs of the distal femur employing a technique recently described in the literatures [17, 18]. In practice, we measured the angle between the cut lines of the posterior femoral condyles and the proximal tibia while patients sat on a table with their lower legs dependent and a 1.5 kg weight attached to the ankle on the treated side, an arrangement which facilitated clear visualization of the shape and width of the flexion gap (Figure 2).

Data are expressed as mean ± standard deviation. We used the ANOVA to identify differences between intraoperative and postoperative gap balances both in extension and flexion. Where differences existed in ANOVA, the Fisher-protected least significant difference test was used to determine significance. We also calculated correlation coefficients between intraoperative and postoperative gap balances both in extension and flexion. The level of significance was set at *P* < 0.05 for all analyses.

This study was approved by the Ethical Committee of Chiba Aoba Municipal Hospital and all patients gave their informed consent before their inclusion in this study.

## 3. Results

### 3.1. Changes in Joint Gap Balances between Intraoperative and Postoperative Measurements

**Figure 3:**
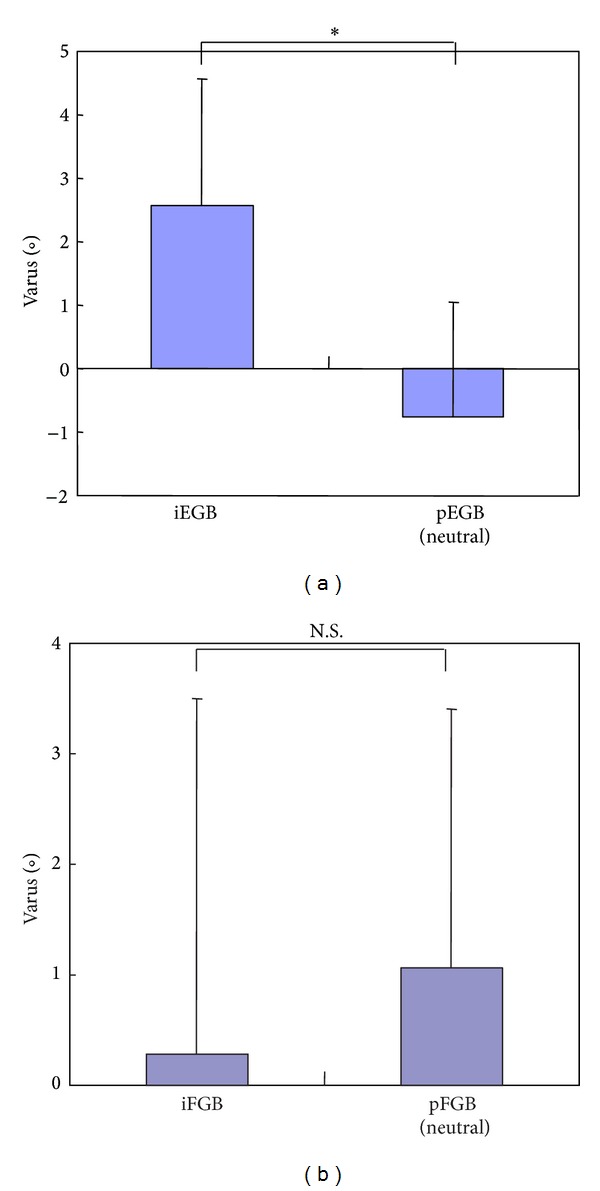
Changes in joint gap balance between intraoperative and postoperative measurements. (a) Intraoperative extension gap balance (iEGB) of 2.6° varus significantly differs from postoperative extension gap balance (pEGB) of 0.77° valgus in neutral position (*P < 0.01). (b) No significant difference is observed between intraoperative flexion gap balance (iFGB) and postoperative flexion gap balance (pFGB) in neutral position.

Intraoperative extension gap balance (iEGB) was 2.6 ± 2.0° varus versus a postoperative extension gap balance (pEGB) in neutral position of 0.77 ± 1.8° valgus, a statistically significant difference (*P* < 0.01) (Figure 3(a)). On the other hand, intraoperative flexion gap balance (iFGB) was 0.29 ± 3.2° varus versus a postoperative flexion gap balance (pFGB) in neutral position of 1.1 ± 2.3° varus, a difference that was not statistically significant (Figure 3(b)).

### 3.2. Correlation between Intraoperative and Postoperative Gap Balance

To evaluate how well intraoperative gap balances correlate with postoperative gap balances, we performed a correlation analysis between iEGB and pEGB and between iFGB and pFGB. However, we observed no significant correlations between both of them (data not shown).

## 4. Discussion

Achieving proper soft tissue balance is one of the most important requirements for successful TKA; however, controversy persists as to whether the intraoperative joint gap in extension should be equal in all aspects to the intraoperative joint gap in flexion.

Sekiya et al. measured coronal, lateral, or medial ligamentous laxity immediately after TKA and at 3, 6, and 12 months postoperatively in 71 knees with preoperative varus deformity and showed that residual lateral ligamentous laxity immediately after surgery subsequently corrected itself spontaneously in some instances [9]. In our study, mean intraoperative gap balance in extension (mean iEGB = 2.6° varus) improved postoperatively to a mean value of 0.77° valgus. We do not know exact reason for this change in alignment but speculate that the improvement of varus balance was caused by the spontaneous correction of lateral ligament laxity as Sekiya et al. reported [9] and/or by the temporally improved medial ligament tightness because we used the CR-type implants in all cases.

The question then arises as to why the flexion gap balance remained stable after TKA. One plausible explanation is that PCL reduces medial ligamentous laxity in flexion both during and after TKA. During TKA for varus knees, the medial collateral ligament is released frequently and osteophytes at the medial femoral chondyle and tibial plateau were removed to enable proper coronal ligament balance, resulting in an increase in medial ligamentous laxity. Thus, as well as the extension gap balance, the flexion gap balance would change to valgus alignment if we used the PS implants. However, we used the CR implants where PCL functioned as a medial supporting stabilizer, which contributed to the stable valance in flexion.

Prior to starting this study, we had hypothesized that intraoperative gap balances would correlate with postoperative gap balances; however, contrary to our expectation, we observed no significant correlation between intraoperative gap balance and postoperative gap balance both in extension and in flexion (data not shown). Possible explanations for this outcome include the following. First, we employed different methods for our intraoperative and postoperative measurements of joint gap balance. Intraoperatively, we distracted the joint gap by 40 lbs and measured the gap balance, whereas we measured the postoperative gap balance with the patient supine on the table without a distraction force. Moreover, we chose 40 lbs as the distraction force based upon a previous study [15], but we do not know how well 40 lbs distraction force reflects physiological conditions of the knee. Second, the operating conditions of the extensor mechanism substantially differ between intraoperative and postoperative environments. Even with the patella in a reduced position during measurements of the intraoperative gap balance, some degree of impairment of the extensor mechanism, especially in flexion, would still persist, which could result in no significant correlation between intraoperative gap balance and postoperative gap balance. Third, we measured the intraoperative tibiofemoral gap balance before implanting femoral components. Because implanting femoral components strongly affects the joint gap balances especially in extension [19], measurements under presence or absence of the femoral components might affect accurate relation between intraoperative gap balance and postoperative gap balance. Due to the relatively small number of TKA cases comprising our study, further studies are needed to more definitively determine how much correlation, if any, there is between intraoperative and postoperative gap balance both in extension and in flexion.

In conclusion, our results showed that intraoperative gap balance was significantly reduced postoperatively in extension but not significantly altered in flexion. Furthermore, we observed no significant correlation between intraoperative gap balance and postoperative gap balance both in extension and flexion. Despite its limitations, our results presented here indicate the importance of avoiding excessive release of the medial soft tissue during TKA for varus knees. Development of surgical procedures to allow accurate predictions of postoperative joint gap balances based upon intraoperative joint gap balances measurements remains a pressing need.
